# [*N*-(3-Meth­oxy-2-oxidobenzyl­idene-κ*O* ^2^)threoninato-κ^2^ *O* ^1^,*N*](1,10-phenanthroline-κ^2^ *N*,*N*′)copper(II) hemihydrate

**DOI:** 10.1107/S1600536811012049

**Published:** 2011-04-07

**Authors:** Buqin Jing, Lianzhi Li, Jianfang Dong, Jinghong Li

**Affiliations:** a College of Chemistry and Chemical Engineering, Shanxi Datong University, Datong, Shanxi 037009, People’s Republic of China; b School of Chemistry and Chemical Engineering, Liaocheng University, Shandong 252059, People’s Republic of China; c Department of Materials Science, Shandong Polytechnic Technician College, Shandong 252027, People’s Republic of China

## Abstract

In the title complex, [Cu(C_12_H_13_NO_5_)(C_12_H_8_N_2_)]·0.5H_2_O, the Cu^II^ ion is five-coordinated by one N atom and two O atoms from a tridentate Schiff base ligand, derived from the condensation of l-threonine and *o*-vanillin, and two N atoms from a 1,10-phenanthroline ligand in a distorted square-pyramidal geometry. In the crystal, inter­molecular O—H⋯O hydrogen bonds form a one-dimensional left-handed helical structureextending parallel to [001]. The water molecule of crystallization shows half-occupancy.

## Related literature

For general background to Schiff bases and their metal complexes, see: Chohan *et al.* (1998 ▶); Nath *et al.* (2001 ▶); Yamada (1966 ▶). For structures of related complexes with five-coordinate copper(II) derived from amino acid Schiff base ligands, see: Huang *et al.* (2010 ▶); Qiu *et al.* (2008 ▶). 
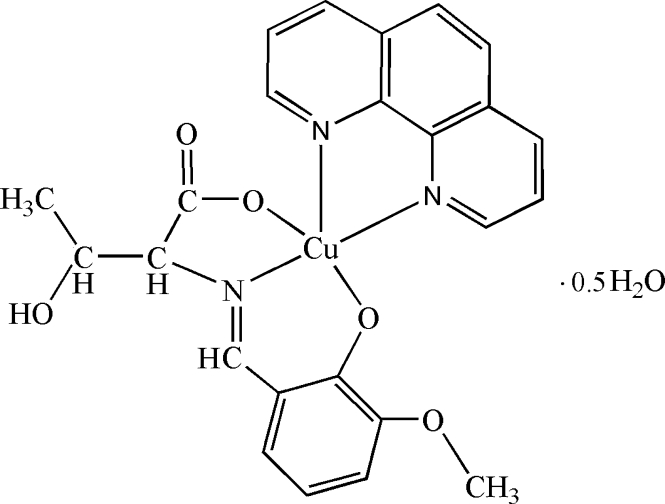


## Experimental

### 

#### Crystal data

[Cu(C_12_H_13_NO_5_)(C_12_H_8_N_2_)]·0.5H_2_O
*M* *_r_* = 503.99
Tetragonal, 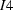

*a* = 22.527 (6) Å
*c* = 10.290 (4) Å
*V* = 5222 (3) Å^3^
*Z* = 8
Mo *K*α radiation
μ = 0.87 mm^−1^
*T* = 293 K
0.50 × 0.15 × 0.11 mm

#### Data collection

Bruker SMART 1000 CCD diffractometer
Absorption correction: multi-scan (*SADABS*; Sheldrick, 1996 ▶) *T*_min_ = 0.669, *T*_max_ = 0.910
13788 measured reflections
4560 independent reflections
2419 reflections with *I* > 2σ(*I*)
*R*_int_ = 0.084

#### Refinement

*R*[*F* ^2^ > 2σ(*F* ^2^)] = 0.076
*wR*(*F* ^2^) = 0.267
*S* = 0.90
4560 reflections
307 parameters
511 restraints
H-atom parameters constrained
Δρ_max_ = 0.83 e Å^−3^
Δρ_min_ = −0.40 e Å^−3^
Absolute structure: Flack (1983 ▶), 2126 Friedel pairs
Flack parameter: −0.13 (5)

### 

Data collection: *SMART* (Bruker, 2007 ▶); cell refinement: *SAINT* (Bruker, 2007 ▶); data reduction: *SAINT*; program(s) used to solve structure: *SHELXS97* (Sheldrick, 2008 ▶); program(s) used to refine structure: *SHELXL97* (Sheldrick, 2008 ▶); molecular graphics: *SHELXTL* (Sheldrick, 2008 ▶); software used to prepare material for publication: *SHELXTL*.

## Supplementary Material

Crystal structure: contains datablocks global, I. DOI: 10.1107/S1600536811012049/hy2419sup1.cif

Structure factors: contains datablocks I. DOI: 10.1107/S1600536811012049/hy2419Isup2.hkl

Additional supplementary materials:  crystallographic information; 3D view; checkCIF report

## Figures and Tables

**Table 1:** Selected bond lengths (Å)

Cu1—N1	1.940 (8)
Cu1—N2	2.263 (9)
Cu1—N3	2.004 (8)
Cu1—O1	1.966 (6)
Cu1—O4	1.924 (7)

**Table 2:** Hydrogen-bond geometry (Å, °)

*D*—H⋯*A*	*D*—H	H⋯*A*	*D*⋯*A*	*D*—H⋯*A*
O3—H3⋯O2^i^	0.82	2.10	2.767 (11)	138

Symmetry code: (i) 
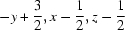
.

**Fig. 1.**
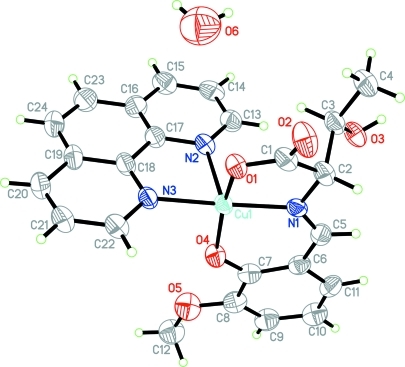
The molecular structure of the title compound, drawn with 30% probability displacement ellipsoids.

**Fig. 2.**
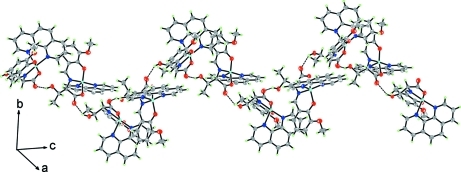
The one-dimensional structure of the title compound, linked by O—H···O hydrogen bonds.

## supplementary crystallographic information

### Comment

Schiff bases still play an important role as ligands in metal coordination chemistry even after almost a century since their discovery (Yamada *et al.*, 1966). It has been reported that amino acid Schiff bases and their first row transition metal complexes exhibit fungicidal, bactericidal, antiviral and antitubercular activities (Chohan *et al.*, 1998; Nath *et al.*, 2001). Herein, we report the synthesis and crystal structure of a new copper(II) complex with a tridentate Schiff base ligand, derived from the condensation of *L*-threonine and *o*-vanillin, and a 1,10-phenanthroline coligand.

The title complex has a mononuclear structure with an amino acid Schiff base ligand and a 1,10-phenanthroline ligand. As shown in Fig. 1, the Cu^II^ ion is five-coordinated by one N atom and two O atoms from the Schiff base ligand and by two N atoms from the 1,10-phenanthroline ligand, forming a seriously distorted square-pyramidal geometry. O1, O4, N1 and N3 atoms are located in the basal plane and N2 atom in the apical position. The Cu^II^ ion lies 0.1651 (4) Å above the basal plane towards N2. The Cu1—N2 bond is significantly longer [2.263 (9) Å] (Table 1) as seen previously [2.298 (4) Å] (Huang *et al.*, 2010) and [2.231 (3) Å] (Qiu *et al.*, 2008). In the crystal, intermolecular O3—H3···O2^i^ (symmetry code: 3/2-y, -1/2+x, -1/2+z) hydrogen bonds (Table 2) form a one-dimensional left-handed helical structure (Fig. 2).

### Experimental

*L*-Threonine (1 mmol, 119 mg) and potassium hydroxide (1 mmol, 56 mg) were dissolved in hot methanol (10 ml) and added successively to a methanol solution of *o*-vanillin (1 mmol, 152 mg). The mixture was then stirred at 323 K for 2 h. Subsequently, an aqueous solution (2 ml) of cupric acetate hydrate (1 mmol, 200 mg) was added dropwise and stirred for 2 h. A methanol solution (5 ml) of phenanthroline (1 mmol, 198 mg) was added dropwise and stirred for 4 h. The solution was held at room temperature for two weeks, whereupon green block-shaped crystals suitable for X-ray diffraction were obtained.

### Refinement

H atoms of the water molecule were found in difference Fourier maps and refined as riding atoms, with O—H = 0.85 Å and with *U*_iso_(H) = 1.5*U*_eq_(O). All other H atoms were placed in geometrically calculated positions and refined as riding atoms, with C—H = 0.93 (aromatic), 0.96 (CH_3_) and 0.98 (CH) Å and with *U*_iso_(H) = 1.2(1.5 for methyl and hydroxyl)*U*_eq_(C, O).

### Crystal data

**Table:**

[Cu(C_12_H_13_NO_5_)(C_12_H_8_N_2_)]·0.5H_2_O	*D*_x_ = 1.282 Mg m^−^^3^
*M**_r_* = 503.99	Mo *K*α radiation, λ = 0.71073 Å
Tetragonal, *I*4	Cell parameters from 1876 reflections
Hall symbol: I 4	θ = 2.2–18.4°
*a* = 22.527 (6) Å	µ = 0.87 mm^−^^1^
*c* = 10.290 (4) Å	*T* = 293 K
*V* = 5222 (3) Å^3^	Block, green
*Z* = 8	0.50 × 0.15 × 0.11 mm
*F*(000) = 2080	

### Data collection

**Table:**

Bruker SMART 1000 CCD diffractometer	4560 independent reflections
Radiation source: fine-focus sealed tube	2419 reflections with *I* > 2σ(*I*)
graphite	*R*_int_ = 0.084
φ and ω scans	θ_max_ = 25.0°, θ_min_ = 1.8°
Absorption correction: multi-scan (*SADABS*; Sheldrick, 1996)	*h* = −26→20
*T*_min_ = 0.669, *T*_max_ = 0.910	*k* = −26→26
13788 measured reflections	*l* = −12→12

### Refinement

**Table:**

Refinement on *F*^2^	Secondary atom site location: difference Fourier map
Least-squares matrix: full	Hydrogen site location: inferred from neighbouring sites
*R*[*F*^2^ > 2σ(*F*^2^)] = 0.076	H-atom parameters constrained
*wR*(*F*^2^) = 0.267	*w* = 1/[σ^2^(*F*_o_^2^) + (0.182*P*)^2^] where *P* = (*F*_o_^2^ + 2*F*_c_^2^)/3
*S* = 0.90	(Δ/σ)_max_ < 0.001
4560 reflections	Δρ_max_ = 0.83 e Å^−^^3^
307 parameters	Δρ_min_ = −0.40 e Å^−^^3^
511 restraints	Absolute structure: Flack (1983), 2126 Friedel pairs
Primary atom site location: structure-invariant direct methods	Flack parameter: −0.13 (5)

### Fractional atomic coordinates and isotropic or equivalent isotropic displacement parameters (Å^2^)

**Table:**

	*x*	*y*	*z*	*U*_iso_*/*U*_eq_	Occ. (<1)
Cu1	0.87663 (4)	0.35499 (4)	0.6986 (2)	0.0480 (4)	
N1	0.9319 (3)	0.3654 (4)	0.5559 (8)	0.0497 (19)	
N2	0.7903 (4)	0.3312 (4)	0.5991 (9)	0.061 (2)	
N3	0.8201 (4)	0.3524 (4)	0.8489 (8)	0.055 (2)	
O1	0.8764 (3)	0.4423 (3)	0.7022 (10)	0.0605 (15)	
O2	0.9201 (4)	0.5211 (3)	0.6114 (8)	0.082 (2)	
O3	0.8996 (3)	0.3845 (3)	0.3014 (7)	0.0669 (19)	
H3	0.9057	0.3985	0.2291	0.100*	
O4	0.8993 (3)	0.2736 (3)	0.7261 (7)	0.061 (2)	
O5	0.9025 (4)	0.1616 (3)	0.7772 (10)	0.089 (3)	
O6	0.0168 (13)	0.2227 (13)	0.067 (3)	0.177 (12)	0.50
H25	0.0133	0.2266	−0.0149	0.265*	0.50
H26	0.0155	0.1854	0.0797	0.265*	0.50
C1	0.9087 (5)	0.4666 (5)	0.6146 (11)	0.063 (2)	
C2	0.9336 (5)	0.4259 (5)	0.5068 (10)	0.063 (2)	
H2	0.9739	0.4376	0.4815	0.075*	
C3	0.8892 (5)	0.4301 (5)	0.3891 (10)	0.065 (2)	
H3A	0.8493	0.4239	0.4244	0.077*	
C4	0.8898 (6)	0.4907 (5)	0.3298 (12)	0.079 (3)	
H4A	0.8622	0.4920	0.2588	0.118*	
H4B	0.9290	0.4995	0.2984	0.118*	
H4C	0.8786	0.5195	0.3940	0.118*	
C5	0.9646 (5)	0.3267 (5)	0.5069 (11)	0.058 (2)	
H5	0.9904	0.3385	0.4414	0.069*	
C6	0.9653 (4)	0.2659 (5)	0.5439 (9)	0.059 (2)	
C7	0.9331 (5)	0.2421 (4)	0.6499 (10)	0.063 (2)	
C8	0.9367 (5)	0.1805 (4)	0.6756 (13)	0.070 (2)	
C9	0.9703 (5)	0.1430 (5)	0.5961 (12)	0.076 (3)	
H9	0.9695	0.1022	0.6104	0.091*	
C10	1.0053 (5)	0.1662 (4)	0.4943 (11)	0.081 (3)	
H10	1.0306	0.1420	0.4465	0.097*	
C11	1.0007 (4)	0.2271 (4)	0.4679 (12)	0.070 (3)	
H11	1.0216	0.2427	0.3979	0.084*	
C12	0.9017 (6)	0.1000 (5)	0.8092 (16)	0.091 (3)	
H12A	0.8767	0.0938	0.8835	0.137*	
H12B	0.9413	0.0870	0.8288	0.137*	
H12C	0.8866	0.0778	0.7368	0.137*	
C13	0.7756 (5)	0.3181 (5)	0.4748 (11)	0.067 (3)	
H13	0.8050	0.3210	0.4116	0.081*	
C14	0.7194 (4)	0.3008 (5)	0.4365 (13)	0.078 (3)	
H14	0.7113	0.2919	0.3500	0.093*	
C15	0.6757 (5)	0.2969 (6)	0.5295 (13)	0.075 (3)	
H15	0.6369	0.2877	0.5055	0.091*	
C16	0.6892 (4)	0.3066 (5)	0.6563 (11)	0.067 (2)	
C17	0.7462 (4)	0.3256 (4)	0.6924 (11)	0.0588 (18)	
C18	0.7628 (5)	0.3374 (5)	0.8214 (8)	0.061 (2)	
C19	0.7194 (5)	0.3332 (5)	0.9200 (8)	0.070 (2)	
C20	0.7350 (5)	0.3438 (5)	1.0495 (10)	0.074 (3)	
H20	0.7076	0.3398	1.1166	0.089*	
C21	0.7923 (5)	0.3605 (6)	1.0725 (11)	0.079 (3)	
H21	0.8035	0.3704	1.1567	0.095*	
C22	0.8342 (5)	0.3630 (5)	0.9741 (10)	0.068 (3)	
H22	0.8733	0.3722	0.9951	0.082*	
C23	0.6463 (6)	0.3046 (6)	0.7582 (11)	0.081 (3)	
H23	0.6072	0.2952	0.7375	0.097*	
C24	0.6605 (5)	0.3161 (6)	0.8868 (11)	0.081 (3)	
H24	0.6317	0.3127	0.9512	0.098*	

### Atomic displacement parameters (Å^2^)

**Table:**

	*U*^11^	*U*^22^	*U*^33^	*U*^12^	*U*^13^	*U*^23^
Cu1	0.0526 (6)	0.0562 (7)	0.0354 (5)	−0.0025 (5)	0.0009 (7)	0.0008 (7)
N1	0.052 (5)	0.059 (5)	0.038 (4)	0.007 (4)	−0.007 (4)	0.001 (4)
N2	0.069 (5)	0.069 (6)	0.046 (5)	0.002 (4)	0.000 (4)	0.009 (4)
N3	0.060 (5)	0.063 (5)	0.041 (5)	−0.003 (4)	0.002 (3)	0.006 (4)
O1	0.071 (4)	0.067 (4)	0.044 (3)	−0.002 (3)	0.004 (5)	0.002 (5)
O2	0.123 (7)	0.055 (4)	0.068 (5)	−0.028 (4)	0.019 (5)	−0.005 (4)
O3	0.093 (5)	0.071 (5)	0.036 (4)	−0.008 (4)	−0.004 (4)	0.000 (3)
O4	0.071 (4)	0.056 (4)	0.055 (6)	−0.002 (3)	0.007 (4)	−0.001 (3)
O5	0.107 (7)	0.051 (4)	0.109 (7)	−0.011 (4)	0.007 (5)	−0.002 (4)
O6	0.155 (13)	0.187 (14)	0.188 (15)	−0.014 (9)	0.013 (9)	−0.002 (9)
C1	0.073 (5)	0.067 (5)	0.050 (5)	−0.002 (5)	−0.005 (5)	0.009 (5)
C2	0.075 (5)	0.069 (5)	0.044 (5)	−0.006 (5)	−0.001 (4)	0.004 (4)
C3	0.075 (5)	0.077 (5)	0.042 (5)	−0.004 (5)	−0.001 (4)	0.010 (5)
C4	0.100 (5)	0.083 (5)	0.054 (5)	−0.001 (5)	0.003 (5)	0.010 (4)
C5	0.053 (5)	0.074 (5)	0.046 (5)	−0.005 (4)	−0.004 (4)	−0.007 (4)
C6	0.053 (5)	0.066 (5)	0.059 (5)	0.004 (4)	−0.010 (4)	−0.006 (4)
C7	0.061 (5)	0.060 (5)	0.068 (5)	0.004 (4)	−0.009 (4)	−0.003 (4)
C8	0.066 (5)	0.065 (5)	0.077 (6)	−0.001 (4)	−0.008 (4)	−0.007 (5)
C9	0.079 (5)	0.062 (5)	0.086 (6)	0.012 (4)	−0.002 (5)	−0.011 (5)
C10	0.080 (6)	0.075 (5)	0.086 (7)	0.007 (5)	−0.006 (5)	−0.014 (5)
C11	0.056 (5)	0.081 (5)	0.073 (6)	0.008 (5)	0.003 (5)	−0.011 (5)
C12	0.099 (5)	0.072 (5)	0.102 (6)	0.001 (5)	−0.002 (5)	0.003 (5)
C13	0.072 (6)	0.076 (6)	0.053 (6)	−0.001 (5)	−0.006 (5)	0.000 (5)
C14	0.085 (6)	0.082 (6)	0.065 (6)	0.000 (5)	−0.026 (5)	0.011 (5)
C15	0.072 (6)	0.081 (6)	0.073 (6)	−0.017 (5)	−0.021 (5)	0.005 (5)
C16	0.058 (4)	0.076 (5)	0.066 (5)	−0.009 (4)	−0.005 (4)	0.001 (4)
C17	0.058 (4)	0.066 (4)	0.052 (4)	−0.008 (3)	−0.002 (4)	0.005 (5)
C18	0.066 (4)	0.070 (5)	0.048 (4)	−0.007 (4)	0.004 (4)	0.010 (4)
C19	0.070 (5)	0.088 (5)	0.052 (5)	−0.001 (5)	0.013 (5)	0.005 (5)
C20	0.080 (6)	0.086 (6)	0.055 (5)	−0.004 (5)	0.016 (5)	0.007 (5)
C21	0.092 (6)	0.096 (6)	0.049 (6)	0.003 (6)	0.002 (5)	0.004 (5)
C22	0.077 (6)	0.071 (6)	0.056 (6)	−0.004 (5)	−0.004 (5)	0.007 (5)
C23	0.067 (5)	0.095 (6)	0.081 (6)	−0.009 (5)	−0.005 (4)	0.010 (5)
C24	0.073 (5)	0.098 (6)	0.073 (6)	−0.006 (5)	0.019 (5)	0.012 (5)

### Geometric parameters (Å, °)

**Table:**

Cu1—N1	1.940 (8)	C6—C11	1.417 (8)
Cu1—N2	2.263 (9)	C7—C8	1.417 (8)
Cu1—N3	2.004 (8)	C8—C9	1.397 (16)
Cu1—O1	1.966 (6)	C9—C10	1.413 (9)
Cu1—O4	1.924 (7)	C9—H9	0.9300
N1—C5	1.246 (13)	C10—C11	1.403 (8)
N1—C2	1.456 (12)	C10—H10	0.9300
N2—C17	1.387 (13)	C11—H11	0.9300
N2—C13	1.354 (14)	C12—H12A	0.9600
N3—C22	1.348 (13)	C12—H12B	0.9600
N3—C18	1.364 (13)	C12—H12C	0.9600
O1—C1	1.282 (13)	C13—C14	1.383 (10)
O2—C1	1.255 (12)	C13—H13	0.9300
O3—C3	1.388 (13)	C14—C15	1.376 (11)
O3—H3	0.8200	C14—H14	0.9300
O4—C7	1.302 (12)	C15—C16	1.358 (17)
O5—C8	1.367 (15)	C15—H15	0.9300
O5—C12	1.426 (13)	C16—C17	1.403 (8)
O6—H25	0.8500	C16—C23	1.427 (16)
O6—H26	0.8501	C17—C18	1.404 (8)
C1—C2	1.544 (15)	C18—C19	1.412 (8)
C2—C3	1.573 (14)	C19—C20	1.398 (8)
C2—H2	0.9800	C19—C24	1.424 (15)
C3—C4	1.494 (15)	C20—C21	1.365 (15)
C3—H3A	0.9800	C20—H20	0.9300
C4—H4A	0.9600	C21—C22	1.386 (10)
C4—H4B	0.9600	C21—H21	0.9300
C4—H4C	0.9600	C22—H22	0.9300
C5—C6	1.422 (15)	C23—C24	1.386 (9)
C5—H5	0.9300	C23—H23	0.9300
C6—C7	1.414 (8)	C24—H24	0.9300
			
N1—Cu1—O4	93.2 (3)	C9—C8—O5	124.4 (9)
N1—Cu1—O1	84.0 (3)	C9—C8—C7	121.0 (11)
O4—Cu1—O1	161.9 (3)	O5—C8—C7	114.5 (10)
N1—Cu1—N3	174.7 (3)	C8—C9—C10	120.8 (11)
O4—Cu1—N3	91.6 (3)	C8—C9—H9	119.6
O1—Cu1—N3	90.8 (3)	C10—C9—H9	119.6
N1—Cu1—N2	103.8 (3)	C11—C10—C9	117.7 (11)
O4—Cu1—N2	94.0 (3)	C11—C10—H10	121.2
O1—Cu1—N2	104.1 (3)	C9—C10—H10	121.2
N3—Cu1—N2	78.2 (4)	C10—C11—C6	122.5 (10)
C5—N1—C2	119.9 (9)	C10—C11—H11	118.8
C5—N1—Cu1	126.9 (8)	C6—C11—H11	118.8
C2—N1—Cu1	113.1 (6)	O5—C12—H12A	109.5
C17—N2—C13	117.4 (9)	O5—C12—H12B	109.5
C17—N2—Cu1	108.9 (6)	H12A—C12—H12B	109.5
C13—N2—Cu1	133.5 (7)	O5—C12—H12C	109.5
C22—N3—C18	117.7 (9)	H12A—C12—H12C	109.5
C22—N3—Cu1	125.6 (7)	H12B—C12—H12C	109.5
C18—N3—Cu1	116.6 (6)	N2—C13—C14	123.6 (11)
C1—O1—Cu1	114.4 (7)	N2—C13—H13	118.2
C3—O3—H3	109.5	C14—C13—H13	118.2
C7—O4—Cu1	125.9 (6)	C15—C14—C13	118.4 (12)
C8—O5—C12	119.1 (10)	C15—C14—H14	120.8
H25—O6—H26	104.6	C13—C14—H14	120.8
O1—C1—O2	123.5 (11)	C14—C15—C16	119.8 (11)
O1—C1—C2	117.2 (9)	C14—C15—H15	120.1
O2—C1—C2	119.2 (10)	C16—C15—H15	120.1
N1—C2—C1	107.3 (8)	C15—C16—C17	120.6 (11)
N1—C2—C3	107.9 (8)	C15—C16—C23	123.3 (9)
C1—C2—C3	106.7 (9)	C17—C16—C23	115.8 (10)
N1—C2—H2	111.6	N2—C17—C16	119.9 (10)
C1—C2—H2	111.6	N2—C17—C18	116.5 (7)
C3—C2—H2	111.6	C16—C17—C18	123.5 (10)
O3—C3—C4	114.2 (9)	N3—C18—C17	119.7 (7)
O3—C3—C2	110.4 (9)	N3—C18—C19	121.4 (9)
C4—C3—C2	111.3 (9)	C17—C18—C19	118.9 (10)
O3—C3—H3A	106.8	C20—C19—C24	120.6 (9)
C4—C3—H3A	106.8	C20—C19—C18	120.0 (10)
C2—C3—H3A	106.8	C24—C19—C18	119.4 (10)
C3—C4—H4A	109.5	C19—C20—C21	116.7 (11)
C3—C4—H4B	109.5	C19—C20—H20	121.6
H4A—C4—H4B	109.5	C21—C20—H20	121.6
C3—C4—H4C	109.5	C22—C21—C20	121.9 (11)
H4A—C4—H4C	109.5	C22—C21—H21	119.1
H4B—C4—H4C	109.5	C20—C21—H21	119.1
N1—C5—C6	124.9 (10)	N3—C22—C21	122.0 (11)
N1—C5—H5	117.6	N3—C22—H22	119.0
C6—C5—H5	117.6	C21—C22—H22	119.0
C7—C6—C11	118.8 (9)	C24—C23—C16	122.6 (11)
C7—C6—C5	124.4 (8)	C24—C23—H23	118.7
C11—C6—C5	116.9 (9)	C16—C23—H23	118.7
O4—C7—C6	123.9 (8)	C23—C24—C19	119.7 (11)
O4—C7—C8	117.1 (9)	C23—C24—H24	120.1
C6—C7—C8	119.0 (10)	C19—C24—H24	120.1

### Hydrogen-bond geometry (Å, °)

**Table:**

*D*—H···*A*	*D*—H	H···*A*	*D*···*A*	*D*—H···*A*
O3—H3···O2^i^	0.82	2.10	2.767 (11)	138

Symmetry codes: (i) −*y*+3/2, *x*−1/2, *z*−1/2.
